# Diagnosis of vascular Ehlers Danlos syndrome and management of vascular complications: a vascular surgeons perspective

**DOI:** 10.1515/medgen-2024-2053

**Published:** 2024-12-03

**Authors:** Laura Schönherr, Sabine Wipper, Yskert von Kodolitsch

**Affiliations:** University Hospital for Vascular Surgery Anichstr. 35 6020 Innsbruck Austria; University Hospital for Vascular Surgery Anichstr. 35 6020 Innsbruck Austria; University Heart & Vascular Center Hamburg Martinistr. 52 20246 Hamburg Germany

**Keywords:** Ehlers, Danlos syndrome, vascular Ehlers, Danlos syndrome, genetic aortic syndromes, genetic connective tissue disorder

## Abstract

The monogenic Ehlers – Danlos syndromes (EDS) constitute a clinically and genetically heterogenous group of connective tissue disorders with overlapping features of generalized joint hypermobility, skin hyperextensibility and tissue fragility. Vascular complications can be observed in several EDS types, but generalized tissue fragility resulting in significant increased risk on vascular events from a young age are a major clinical characteristic of vascular Ehlers – Danlos (vEDS, former Type IV). This is a rare, monogenic EDS type, with a suspected prevalence of 1:50 000. Even though progress regarding awareness and management of vEDS has been made, further studies are needed regarding optimal treatment and follow up. In this manuscript we present the perspective of a vascular surgeon regarding the current literature to management and treatment options for vascular complications in vEDS.

## Introduction

The monogenic Ehlers – Danlos syndromes (EDS) constitute a heterogenous group of genetic connective tissue disorders (CTD), with currently 12 known types. They have overlapping features of generalized joint hypermobility, skin hyperextensibility and generalized tissue friability but also have their own distinctive clinical manifestations [1]. Major vascular complications have been observed in several EDS types including kyphoscoliotic EDS (kEDS) where medium sized arterial aneurysms are part of the minor diagnostic criteria. However, the frequency of vascular complications differs significantly, with life threatening vascular events such as aneurysm formation or arterial dissection being most prevalent in vascular EDS (vEDS) [2, 3].

This is a rare, monogenic EDS type that affects approximately 5 % of all individuals diagnosed with EDS [3]. It is caused by heterozygous pathogenic variants in the *COL3A1* gene, which leads to a significant reduction in the production of type III (pro)collagen, resulting in generalized tissue friability [4]. Clinical manifestations of vEDS primarily involve medium – sized arteries, where patients are prone to arterial dissection, aneurysm formation and rupture. Additionally, spontaneous hollow organ perforation such as bowel or less frequently, uterine rupture, can be observed and are part of the major diagnostic criteria [2]. Traditionally, there has been a bias in publications reporting on patients with more severe vascular complications leading to a median life expectancy of 51 years and a complication rate as high as 80 % by age 40 [2, 5]. However, recent studies by Frank et al. and Bowen et al. have reported lower complication rates, likely due to more extensive (predictive) genetic testing leading also to the identification of individuals with milder clinical features and recognition of significant intra- and interfamilial variability in occurrence and severity of symptoms [6, 7].

## Diagnosis of vascular Ehlers – Danlos syndrome

Diagnosing vEDS can be challenging due to its clinically variable presentation and clinical overlap with other inherited connective tissue disorders. This raises the important question for clinicians: when should vEDS be suspected?

According to the 2017 International Classification of Ehlers-Danlos Syndromes, major and minor diagnostic criteria for vEDS were established and are outlined in Table 1. In case of strong clinical suspicions on vEDS further referral to a medical geneticist is indicated, however all clinicians can send blood samples (EDTA tube) for further genetic testing. DNA storage is also possible in urgent situations, and consent to inititate genetic testing can be obtained at a later stage. Genetic testing is required to confirm the suspected diagnosis of vEDS. A recent study by Yamaguchi et al. highlighted the effectiveness of next-generation sequencing (NGS) in diagnosing vEDS. The study demonstrated that NGS, combined with copy number variation (CNV) analysis, accurately detected a wide range of pathogenic *COL3A1* variants, in patients with both typical and atypical presentations of vEDS. Importantly, the study confirmed the presence of *COL3A1* variants in patients initially suspected of having other connective tissue disorders, such as Loeys-Dietz syndrome, emphasizing the clinical overlap between these conditions [8]. Notably, the *COL3A1* gene is also routinely included in the heritable thoracic aortic disease (HTAD) gene panels [9]. To provide further guidance for vascular surgeons in daily practice the following flowchart has been established.

**Table 1:** Major and minor criteria in vascular Ehlers – Danlos syndrome [10]

Major criteria	Minor criteria
Family history of vEDS with documented gene mutation *COL3A1*	Spontaneous bruising
Arterial rupture at young age	Thin, translucent skin with increased venous visibility
Spontaneous sigmoid colon perforation with no underlying other disease	Characteristic facial appearance
Uterine rupture during the third trimester with no previous C-section and/or severe peripartum perineum tears	Spontaneous pneumothorax
Carotido-cavernous sinus fistula without prior trauma	Acrogeria
	Talipes equinovarus
	Congenital hip dislocation
	Hypermobility of small joints
	Tendon and muscle rupture
	Keratoconus
	Gingival recession and gingival fragility
	Early onset varicose veins <30yrs. & nulliparous if female

**Figure 1:**
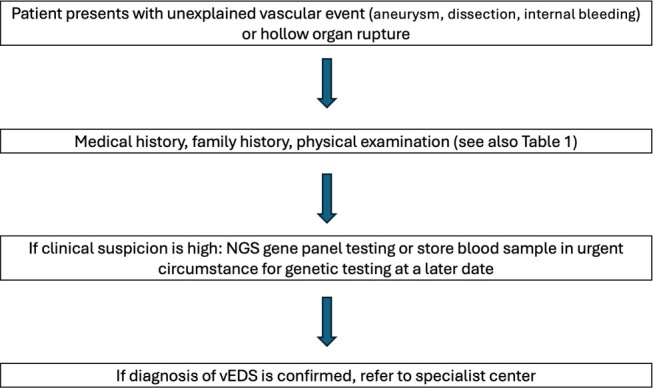
Diagnostic flowchart from a vascular surgeons perspective

## Follow up

**Figure 2:**
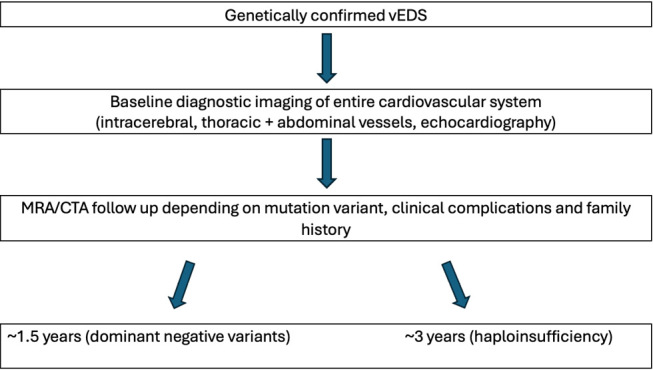
Follow up imaging strategies based on data by the Eurfopean Network for Rare Vascular Diseases (VASCERN)

For patients with genetically confirmed vEDS, no universally accepted evidence-based follow-up or surveillance strategies are currently in place [11, 12]. However, multidisciplinary management is recommended, involving a team of primary care physicians, medical geneticists, vascular-, orthopedic- and general surgeons (depending on clinical manifestations), ideally within specialized centers [2, 11]. The European Vascular Surgery Guidelines emphasize the importance of baseline diagnostic imaging, which includes the entire cardiovascular system (intracerebral, thoracic, and abdominal vessels) as well as transthoracic echocardiography. Frequency and extent of follow-up imaging should be personalized based on individual clinical features and complications [11]. The European Network for Rare Vascular Diseases (VASCERN), which evaluated current strategies for monitoring and surveillance of vEDS patients across expert centers in Europe, recommends non-invasive imaging methods such as Magnetic Resonance Angiography (MRA) and Computed Tomography Angiography (CTA) as the preferred modalities. Monitoring intervals typically range from annually to every five years, with most centers adopting a schedule of 1.5 to 3 years depending on clinical features, including the patient’s specific *COL3A1* variant (see Figure 2). For patients with common glycine substitutions and splice site variants leading to a dominant negative effect and 87.5 % decrease of (pro)collagen type III production, shorter intervals are recommended. While those with variants leading to haploinsufficiency (50 % reduction of (pro)collagen type III) with a complete deletion of one of the two *COL3A1* alleles, are monitored less frequently due to their generally milder phenotype with improved life expectancy (close to that of the general US population) and significantly fewer obstetric and bowel complications [13]. Regular arterial monitoring is considered important for detecting silent arterial events, though the precise benefit of early detection in reducing mortality has yet to be fully established. Continued research reporting accurately on natural history of large cohorts with genetically confirmed vEDS are needed to optimize surveillance protocols and better understand their impact on patient out-comes [12].

## Medical and surgical treatment options for vEDS

Data on optimal medical treatment for vEDS remains limited. A 2010 randomized trial (BBEST trial) including 53 individuals of which 33 had a molecular confirmation of vEDS, demonstrated that Celiprolol, a cardioselective beta-blocker, reduces vascular events in vEDS by providing more stable hemodynamic conditions leading to a less fragile arterial wall [14, 15]. A subsequent study by Baderkhan et al., confirmed its protective effect in 40 genetically confirmed vEDS patients with an annual risk of a major vascular event of 4.7 %, similar to the treatment arm of the BBEST trial (5 %) and lower than in the control arm of the same study (12 %) [15]. However, a recent 12-year study from Italy reported that, despite Celiprolol therapy, vEDS patients still face a yearly risk of 8.8 % for symptomatic vascular events and 6.4 % for life-threatening events, even at the maximum dose of 400 mg of Celiprolol daily [6, 16].

While Celiprolol reduces the risk for major vascular complications, it does not eliminate them, highlighting the need for additional therapeutic strategies.

Research involving mouse models of vEDS suggested beneficial properties of Angiotensin II receptor blockers (ARBs) and pharmacologic inhibitors of ERK1/2 or PKCβ signaling [17]. These results may offer future benefits for vEDS patients, although further studies in cohorts of individuals with molecularly confirmed vEDS are needed to validate their efficacy [18]. To summarize, guidelines currently recommend 400 mg of Celiprolol daily for all confirmed vEDS patients [11, 19]. However, combined therapy with beta blocker and angiotensin II receptor blocker also appeared to lead to a reduction in vascular events [7].

Data on surgical treatment for vEDS is also scarce [11, 20]. Due to the high risk of complications, surgical interventions should ideally be performed only when absolutely necessary and at specialized centers with expertise in managing tissue friability. Knowing that an individual has vEDS in advance of surgery is of great importance as it allows the surgical team to prepare accordingly. To support clinicians the European Network for Rare Vascular Diseases (VASCERN) has published a comprehensive “Do’s and Don’ts” factsheet to guide physicians in managing the various complications associated with vEDS. This resource offers practical recommendations and outlines potential interventions to avoid, helping healthcare professionals provide safer and more effective care for vEDS patients [21]. Given the extreme fragility of blood vessels in vEDS, decisions regarding surgical intervention of vascular complications must be made cautiously. There are no recommendations for prophylactic repair of aortic or vascular dilatations currently in the guidelines [2, 19, 20]. Open surgical repair remains the primary approach for vascular complications, though specialized techniques – such as avoiding vascular clamps or using protective soft covers, along with circumferential reinforcement of anastomosis and tensionless reconstruction – are critical to minimizing complications [9, 22, 23]. While endovascular treatments, such as coil embolization, may be considered, the use of stent grafts remains controversial due to limited data on their long-term durability in vEDS, especially given the weakened vessel walls [4, 9, 24]. Recent reports suggest that endovascular repair may be viable in select vEDS patients, particularly those with null* COL3A1* variants, which are associated with milder disease phenotypes. In these cases, null variants lead to less severely reduced production of (pro)collagen type III, potentially improving surgical outcomes in this group [24]. However, larger studies are required to establish the safety and long-term efficacy of this approach. Pregnant women with vEDS are at a higher risk of complications such as uterine rupture and major aortic events, with a maternal mortality rate of around 5.3 %. As a result, these patients require specialized obstetric care in centers with a vascular surgery team, frequent imaging and elective cesarean section at 34 weeks readily available in case of complications [7].

Overall, the literature supports a conservative approach recommending invasive procedures to be avoided whenever possible. Clinically stable patients should be managed conservatively with close monitoring, as aggressive surgical intervention may increase the risk of complications rather than prevent them [11, 19, 20].

## Conclusion

The management of vascular complications, especially major vascular events, in vEDS remains a significant challenge for clinicians. Due to the rarity of the disease and the lack of large datasets, optimal management requires multidisciplinary teams with specialized knowledge of vEDS to maximize treatment options and bail-out strategies. Early referral to specialized centers equipped with such teams may improve the chances of early diagnosis, better surveillance, and more comprehensive care. Additionally, genetic testing should be an integral part of the diagnostic and management process to guide personalized treatment decisions. Given the limited number of patients, further studies are needed to explore the role of medical therapies—such as ARBs and PKCβ/PI3K/AKT inhibitors—as well as optimal surgical and endovascular treatment options. These advancements, alongside individualized care plans, may ultimately improve outcomes for patients with vEDS.
